# Human dental pulp stem cells attenuate streptozotocin-induced parotid gland injury in rats

**DOI:** 10.1186/s13287-021-02646-6

**Published:** 2021-11-14

**Authors:** Rasha H. Al-Serwi, Ahmed Othman Fathy Othman El-Kersh, Gehan El-Akabawy

**Affiliations:** 1 Oral Basic Sciences, College of Dentistry, Princess Nourah Bint Abdulrahman University, Riyadh, Saudi Arabia; 2 Oral Biology Department, Faculty of Dentistry, Mansoura University, Mansoura, Egypt; 3 Faculty of Dentistry, Kafr El Sheikh University, Kafr El Sheikh, Egypt; 4 Department of Basic Sciences, College of Medicine, Princess Nourah Bint Abdulrahman University, Riyadh, Saudi Arabia; 5 Department of Anatomy and Embryology, Faculty of Medicine, Menoufia University, Menoufia, Egypt

**Keywords:** Dental pulp stem cells, Diabetes, Streptozotocin, Parotid gland

## Abstract

**Objective:**

Diabetes mellitus causes deterioration in the body, including serious damage of the oral cavity related to salivary gland dysfunction, characterised by hyposalivation and xerostomia. Human dental pulp stem cells (hDPSCs) represent a promising therapy source, due to the easy, minimally invasive surgical access to these cells and their high proliferative capacity. It was previously reported that the trophic support mediated by these cells can rescue the functional and structural alterations of damaged salivary glands. However, potential differentiation and paracrine effects of hDPSCs in diabetic-induced parotid gland damage have not been investigated. Our study aimed to investigate the therapeutic effects of intravenous transplantation of hDPSCs on parotid gland injury in a rat model of streptozotocin (STZ)-induced type 1 diabetes.

**Methods:**

Thirty Sprague–Dawley male rats were randomly categorised into three groups: control, diabetic (STZ), and transplanted (STZ + hDPSCs). The hDPSCs or the vehicles were injected into the rats’ tail veins, 7 days after STZ injection. Fasting blood glucose levels were monitored weekly. A glucose tolerance test was performed, and the parotid gland weight, salivary flow rate, oxidative stress indices, parotid gland histology, and caspase-3, vascular endothelial growth factor, proliferating cell nuclear antigen, neuronal nitric oxide synthase, endothelial nitric oxide synthase, and tetrahydrobiopterin biosynthetic enzyme expression levels in parotid tissues were assessed 28 days post-transplantation.

**Results:**

Transplantation of hDPSCs decreased blood glucose, improved parotid gland weight and salivary flow rate, and reduced oxidative stress. The cells migrated to the STZ-injured parotid gland and differentiated into acinar, ductal, and myoepithelial cells. Moreover, hDPSCs downregulated the expression of caspase-3 and upregulated the expression of vascular endothelial growth factor and proliferating cell nuclear antigen, likely exerting pro-angiogenic and anti-apoptotic effects and promoting endogenous regeneration. In addition, the transplanted cells enhanced the parotid nitric oxide-tetrahydrobiopterin pathway.

**Conclusions:**

Our results showed that hDPSCs migrated to and survived within the STZ-injured parotid gland, where functional and morphological damage was prevented due to the restoration of normal glucose levels, differentiation into parotid cell populations, and stimulation of paracrine-mediated regeneration. Thus, hDPSCs may have potential in the treatment of diabetes-induced parotid gland injury.

**Supplementary Information:**

The online version contains supplementary material available at 10.1186/s13287-021-02646-6.

## Introduction

Diabetes mellitus affects almost all organs and organ systems of the body and causes significant damage in the oral cavity [1–4]. The majority of oral changes related to salivary gland dysfunction are characterised by hyposalivation and xerostomia, which significantly reduce the quality of life in patients with diabetes [5–7]. Both basal and stimulated secretion of the parotid and submandibular glands are dramatically downregulated under prolonged hyperglycaemia in humans and rats [8–11].

Diabetes is accompanied by atrophy of the parotid and submandibular salivary glands, as evidenced by reductions in gland weight and size, resulting from degeneration of acinar cells and intralobular ducts [10–18]. These salivary gland changes are likely caused by diabetes-induced oxidative damage. Several studies have reported that the parotid glands are more vulnerable to both antioxidant disturbances and oxidative damage, demonstrating prominent degenerative changes [10, 11, 17, 19, 20]. Indeed, upon stimulation, the parotid gland, one of the main salivary glands, secretes more than 50% of the total saliva [21].

Mesenchymal stem cells (MSCs) have shown promising therapeutic potential in different diseases and tissue restoration. Adult stem cells have been isolated from numerous organs in the body and differentiated into specific cell lineages. Their transplantation carries a negligible risk of tumourigenesis and raises limited ethical concerns [22]. MSCs have been isolated from bone, adipose, and lung tissues as well as from the umbilical cord and dental pulp [23]. MSC-based therapies for atrophic salivary gland have succeeded in decelerating the degeneration process in irradiation-injured salivary glands [24–26], Sjögren’s syndrome [27–30], and diabetes [31]. MSCs can differentiate into acinar and duct cells and exhibit paracrine effects by releasing anti-inflammatory, vasculogenic, and antioxidative or anti-apoptotic factors. Furthermore, MSCs are hypoimmunogenic, because they lack the expression of major histocompatibility complex (MHC) II and express low levels of MHC I [32].

Dental pulp stem cells (DPSCs) are multipotent MSCs that have been demonstrated to be useful not only for the treatment of oral cavity diseases but also for diverse systemic regenerative applications. Several studies suggest that DPSCs have therapeutic potential for the treatment of many diseases, such as Alzheimer’s disease, Parkinson’s disease, cerebral ischemia, spinal cord injuries, myocardial infarction, muscular dystrophy, liver diseases, and immune and eye-related disorders [33–39]. DPSCs have attracted considerable attention as a promising stem cell source for clinical application, owing to their easy obtainability, less invasive collection procedures, limited ethical concerns, high proliferation capacity, and applicability in autologous therapy [40].

Previous studies have shown that the trophic support provided by DPSCs maintains the proliferation and survival of human salivary gland (HSG) cell lines and primary salivary glands [41]. Moreover, the angiogenic role of DPSCs has been demonstrated in diabetes [42, 43] and irradiation-induced injury [44]. However, there are similarities in the development of the tooth bud and the parotid salivary gland, because they both originate from the ectoderm, and between the epithelial-mesenchymal molecular interactions of the tooth bud and the salivary glands [41, 45]; thus, DPSCs may have the potential to differentiate into parotid gland cells if the proper niche is provided. In addition, the potential paracrine effects of DPSCs in diabetes-induced parotid gland damage have not been investigated.

The aim of this study was to investigate whether intravenously administered human DPSCs (hDPSCs) ameliorate hyperglycaemia, migrate to the damaged parotid gland, differentiate into acinar and duct cells, and improve parotid gland morphology. In addition, we evaluated the anti-apoptotic and angiogenic abilities of these cells to gain insights into the mechanisms underlying their therapeutic effects.

## Methods and materials

### Animals

Thirty Sprague–Dawley male rats (aged 6–8 weeks, 150–200 g) were purchased from the Theodor Bilharz Research Institute, Imbaba, Egypt, and maintained in the animal house of the Faculty of Medicine, Menoufia University, Egypt. The rats were group-housed (four per cage) in standard polycarbonate cages under standard laboratory conditions (temperature 22 °C ± 5 °C, humidity 60% ± 5%, and a 12:12-h daylight/darkness cycle). Standard laboratory chow and tap water were available ad libitum.

### Isolation and culture of hDPSCs

Normal human impacted third molars were extracted from healthy adult patients at the Oral Surgery Department, Faculty of Dentistry, Mansoura University, Egypt, and rinsed with Dulbecco's modified Eagle medium (DMEM; Gibco, Carlsbad, CA, USA) supplemented with 3% penicillin–streptomycin (Gibco), immediately after retrieval. All procedures were approved by the Research Ethics Committee of the Faculty of Dentistry, Mansoura University, Egypt, and conducted after obtaining informed consent from all the participants. The hDPSCs were isolated and cultured as previously described [46].

Briefly, the pulp tissues were immediately separated from the teeth and digested in a solution of 3 mg/mL collagenase type 1 (Sigma-Aldrich, St. Louis, MO, USA) for 1 h at 37 °C. The cells (1 × 10^5^ cells/well) were seeded in 6-well cell culture plates (Falcon); cultured in DMEM supplemented with 20% foetal bovine serum (FBS, Gibco), 100 U/mL penicillin, and 100 μg/mL streptomycin; and maintained at 37 °C in a humidified incubator with 5% CO_2_. The culture medium was changed every 3 days. After reaching 70–80% confluence, the cells were trypsinised using 0.05% trypsin–EDTA (Sigma-Aldrich) for 2–5 min and then neutralised by adding complete medium. The detached cells were centrifuged at 500 × *g* for 5 min, and the cell pellet was resuspended in complete medium. To assess cell viability, equal volumes of the cell suspension and 0.4% Trypan blue (Gibco) were mixed, and 10 µL of the prepared sample was loaded in each chamber of a haemocytometer. Viable and nonviable cells were counted within 5 min of preparing the samples. Cell suspensions with more than 90% viability were subcultured at a 1:3 ratio (passage 1). Cells were used at passage 4.

### Induction of diabetes and blood glucose monitoring

Diabetes was induced using intraperitoneal injections of 50 mg/kg streptozotocin (Sigma-Aldrich). STZ-induced diabetes is a widely used rodent model of type I diabetes. STZ was dissolved in a freshly prepared 0.1 M citrate buffer (pH 4.5). The sample size was calculated using G Power software [47]. Twenty rats were fasted for 12 h before STZ injection. After STZ injection, the rats were provided with standard laboratory chow and 10% sucrose water for 48 h to prevent fatal hypoglycaemia and closely monitored every 2 h for 12 h for marked hypoactivity, unresponsiveness, or convulsions. Fasting blood glucose levels were measured 72 h after STZ injection using blood drawn from the retro-orbital plexus of conscious rats. The blood glucose concentrations were determined using a Span Diagnostic Kit with Jinque test strips. Rats with blood glucose levels above 250 mg/dL were considered diabetic. Diabetic rats were randomly distributed into two groups: diabetic (STZ) and transplanted (STZ + hDPSCs) (*n* = 10 for each group). Ten rats received vehicle (citrate buffer) only and served as the control group. The fasting blood glucose levels were measured between 8:00 and 10:00 a.m. once weekly from the day of transplantation (day 0) to day 28.

Glucose tolerance tests were performed 28 days post-transplantation. After a 12-h fast, the rats were injected intraperitoneally with a glucose solution (2 g/kg body weight). Serum blood glucose levels were measured 0, 30, 60, 120, 180, and 240 min after the glucose injection.

### Flow cytometry

Cells were resuspended in 2% FBS/phosphate-buffered saline (PBS), and cell surface staining was performed using fluorescein isothiocyanate-conjugated mouse anti-human CD105, fluorescein isothiocyanate-conjugated mouse anti-human CD90, fluorescein isothiocyanate-conjugated mouse anti-human CD146, phycoerythrin-conjugated mouse anti-human CD29, phycoerythrin-conjugated mouse anti-human CD34 (BD Pharmingen, USA), or phycoerythrin-conjugated mouse anti-human stromal precursor antigen-1 (STRO-1; Invitrogen, USA) antibodies for 30 min at 4 °C. Isotype-identical antibodies served as controls. The cells were analysed using a Beckman Coulter EPICS XL flow cytometer.

### Cell transplantation

One week after the STZ injections, the rats of the STZ + hDPSCs group were injected intravenously into the tail vein with 100 μL serum-free DMEM containing 1 × 10^6^ hDPSCs labelled with the membrane-bound fluorescent marker PKH26 (Sigma-Aldrich). Control and STZ rats received an intravenous injection of serum-free DMEM.

### In vitro multi-differentiation capacity

Adipogenic differentiation was induced using a medium that consisted of DMEM supplemented with 10% FBS, dexamethasone (100 nM), 3-isobutylmethylxanthine (0.1 mM), indomethacin (0.25 mM), and insulin (10 μM) (Sigma-Aldrich). The medium was changed twice a week for 2 weeks. Cells were washed with PBS, fixed in 10% formalin, and stained with 0.3% Oil-Red O (Sigma-Aldrich) in 60% isopropanol reagent.

For osteogenic induction, hDPSCs were cultured in DMEM supplemented with 10% FBS, ascorbate 2-phosphate (200 μM), β-glycerophosphate (10 mM), and dexamethasone (10 nM) (Sigma-Aldrich). The medium was changed twice a week for 2 weeks. Cells were washed with PBS, fixed with 10% formalin, and stained with 2% Alizarin red S (Sigma-Aldrich).

### Assessment of oxidative stress and antioxidant indices

The extent of lipid peroxidation was assessed by measuring the malondialdehyde (MDA) concentration. Trichloroacetic acid (20%) was added to the homogenate and then centrifuged at 5000 × *g* for 15 min. The supernatant was collected, and 0.5% thiobarbituric acid solution was added. After boiling for 10 min in a water bath, the absorbance was measured at 532 nm. The concentration of MDA was calculated using a standard curve, and the results were expressed as nM per mg of protein.

The superoxide dismutase (SOD) activity was measured based on the inhibition of nitroblue tetrazolium reduction by the O_2_ generated by the xanthine/xanthine oxidase system. The absorbance was measured at 550 nm. One SOD activity unit was defined as the enzyme concentration necessary to cause 50% inhibition in 1 mL reaction solution per mg of tissue protein, and the results were expressed as units (U) per mg of protein.

The total antioxidant capacity (TAS) level was evaluated using a kit supplied by Randox (Crumlin, UK). ABTS (2,2′-binamine-di-3-ethylbenzothiazolin-6-sulphonic acid) was incubated with peroxidase (metmyoglobin) and hydrogen peroxide to produce the cationic ABTS^+^ of blue-green colour. The colour was measured spectrophotometrically at 600 nm. The reduction in ABTS^+^ production and the subsequent decrease in colour intensity is proportional to the concentration of antioxidants.

The total oxidative status (TOS) was assessed using a commercial kit (PerOx, TOS/TOC) supplied by Immune Diagnostic (Bensheim, Germany). Peroxidase reacts with peroxides in the sample followed by the conversion of 3,3′,5,5′-tetramethylbenzidine to a coloured product. The reaction was stopped by the addition of the stop solution, which led to a change in colour. A microtiter plate reader was used to measure the absorbance of the sample at 450 nm. The quantification was performed by using the calibrator.

### Salivary flow rate (SFR) measurement

To assess the secretory function of the salivary glands weekly, rats of different groups were anaesthetised by intraperitoneal injection with pentobarbital (80 mg/kg body weight). Then, salivation was stimulated with an intraperitoneal injection of pilocarpine nitrate (5 mg/kg body weight, Sigma-Aldrich). After 5 min of secretion stimulation, total saliva was collected using preweighed cotton swabs placed at the bottom of the oral cavity for up to 15 min, and the quantity of saliva collected was calculated by subtracting the weight of the cotton swabs before and after collection. The saliva weight was divided by the time of duration of the collection (15 min) and the flow rate was calculated in µL/min, which is equivalent to mg/min, since over 99% of the saliva consists of water.

### Real-time quantitative polymerase chain reaction (RT-qPCR) analysis

Total RNA was isolated from homogenised parotid glands of each group using RNeasy Purification Reagent (Qiagen, Valencia, CA, USA), according to the manufacturer’s instructions. The purity of total RNA was assessed with a spectrophotometer and the wavelength absorption ratio (260/280 nm) was between 1.8 and 2.0 for all preparations. Reverse transcription of total RNA to cDNA was carried out using reverse transcription reaction (Superscript II, Gibco Life Technologies, Grand Island, NY, USA). Real-time PCR amplification and analysis were carried out using the StepOne software (version 3.1, Applied Biosystems, Foster City, CA, USA). The reaction mixture contained SYBR Green Master Mix (Applied Biosystems), gene-specific primer pairs (shown in Table 1), cDNA, and nuclease-free water. The cycling conditions were 10 min at 95 °C followed by 40 cycles of 15 s at 95 °C and 60 s at 60 °C. Data were analysed using the ABI Prism sequence detection system software and quantified using the v1·7 Sequence Detection Software from Applied Biosystems. The relative expression of the genes was calculated using the comparative cycle threshold method. All values were normalised to β-actin mRNA which was used as an internal control in all samples.

**Table 1:** List of primers used in RT-qPCR

Gene name	Primer Sequence Forward / Reverse 5' → 3'
eNOS	ATT GCA CCC TTC CGG GGA TT ACG GTT TGC AGG ACG CTG GTT
nNOS	GAA TAC CAG CTG ATC CAT GGA AC TCC TCC AGG AGG GTG TCC ACC GCA TG
BETA ACTIN	ATT TGG CAC CAC ACT TTC TAC A TCA CGC ACG ATT TCC CTC TCA G

### Western blot analysis

Proteins from the parotid tissues were extracted with radioimmunoprecipitation buffer (Sigma-Aldrich) with protease and phosphatase inhibitors. The obtained homogenates were centrifuged at 12,000 × *g* and 4 °C for 20 min; then, the protein concentration was quantified in lysate aliquots using a protein assay kit (Bio-Rad, Hercules, CA, USA). After boiling at 95 °C for 5 min, samples (20 µg/lane) were separated using 7% sodium dodecyl sulphate–polyacrylamide gel electrophoresis and then transferred to nitrocellulose membranes (Bio-Rad). The membranes were blocked using 5% bovine serum albumin in tris-buffered saline (TBS) for 1 h at RT, then incubated overnight at 4 °C with primary antibodies specific for anti-dihydrofolate reductase (DHFR, 1:500, rabbit monoclonal, Abcam, Cambridge, UK). The membranes were washed with TBS. and then incubated with a secondary horseradish peroxidase-conjugated anti-rabbit IgG antibody (1: 5000, Bio-Rad) for 1 h at RT, followed by washing with TBS. Proteins were detected by enhanced chemiluminescence (ECL plus; Amersham, Arlington Heights, IL, USA) and quantified using densitometry and Molecular Analyst Software (Bio-Rad). The relative expression of each protein band was normalised to β-actin.

### Immunohistochemistry and immunofluorescence analysis

At the end of the experiment, each rat was anaesthetised using an intraperitoneal injection of ketamine (90 mg/kg) and xylazine (15 mg/kg), and decapitated. Parotid glands were immediately dissected and weighed. For histological staining, the parotid glands were fixed in 10% formalin and embedded in paraffin wax. Then, 5-μm-thick sections were deparaffinised, rehydrated, and rinsed with PBS and blocked for 30 min in 0.1% H_2_O_2_ to inhibit endogenous peroxidase activity. After rinsing with PBS, the sections were incubated for 1 h in blocking solution (10% normal goat serum) at RT. The sections were then incubated at RT for 1 h with the following primary antibodies: rabbit anti-vascular endothelial growth factor (VEGF; 1:500; cat. No. RB-222; LabVision, Fremont, CA, USA), rabbit anti-proliferating cell nuclear antigen (PCNA; 1:500; cat. No. ab18197; Abcam, Cambridge, UK), and rabbit anti-caspase-3 (1:500: cat. No. ab4051; Abcam). Subsequently, the sections were rinsed with PBS and incubated with the secondary biotinylated goat anti-rabbit antibody (1:200; cat. No. BA-1000; Vector laboratories, Peterborough, UK) at RT for 20 min. Next, the sections were rinsed with PBS and incubated with the enzyme conjugate streptavidin–horseradish peroxidase solution for 10 min. After rinsing in PBS, the sections were developed using 3,3-diaminobenzoic acid (DAB) dissolved in PBS along with H_2_O_2_ (0.03%) immediately before use. Then, the sections were rinsed with PBS, counterstained using two drops (100 μL) of haematoxylin, and washed in distilled water until they turned blue. Finally, the slides were dehydrated using an ascending graded ethanol series (70%, 95%, and 100%) for 5 min per concentration, cleared in xylene, mounted with Histomount, and covered with a coverslip.

For immunofluorescence staining, parotid glands were excised and postfixed for 24 h at 4 °C before being cryoprotected in 30% sucrose at 4 °C. Serial Sects. (40 μm) were cut using a cryostat and stored at − 20 °C. The sections were incubated for 1 h in 10% blocking solution (10% normal goat serum in 0.3% Triton X-100 in PBS) at RT, followed by incubation overnight at 4 °C with the following primary antibodies: rabbit anti-aquaporin 5 (AQP5; 1:1000; cat. No. ab78486; Abcam), rabbit anti-cytokeratin 7 (CK7; 1:200: cat. No. ab18159; Abcam), or rabbit anti-α-smooth muscle actin (α-SMA; 1:200; cat. No. ab5694; Abcam). The sections were washed with PBS and incubated with the appropriate secondary antibody (1:500; AlexaFluor 488; cat. No. A-11034; Molecular Probes, Eugene, OR, USA) for 1 h at RT. Finally, the sections were rinsed with PBS and mounted using Vectashield with 4′,6-diamidino-2-phenylindole (DAPI) (Vector Laboratories). The reliability of PKH26 labelling was assessed against a mouse anti-human nuclei antibody (HNA; 1:400: cat. No. MAB1282; Merck) (Additional file 1: Fig. S1). The specificity of the antibodies used in the study was evaluated (Additional file 1: Figs. S2-S7).

### Quantitative histological assessments

For the quantitative assessment of immunostaining, three nonoverlapping images were randomly captured per section using a Leica DML B2/11888111 microscope equipped with a Leica DFC450 camera and Leica C PLAN 10 × 0.22 objective or a Leica DM5500 B/11888817/12 microscope equipped with a Leica DFC450C camera and Leica HI PLAN 10 × 0.25 objective. For each image, the field of view was at 10 × magnification. The number of immunopositive cells in the fields from at least three sections/animal was counted using ImageJ software (US National Institutes of Health, Bethesda, MD, USA) and averaged per field for each animal. This was blindly performed by an independent, experienced researcher. The calculated percentages for at least eight animals per experimental group were considered for comparison and statistical analyses.

### Statistical analysis

The data are expressed as the mean ± SEM. Normal distributions were evaluated using the D'Agostino and Pearson Omnibus normality test, and data were analysed using one-way or two-way analysis of variance (ANOVA) followed by a post hoc Bonferroni test. *P* < 0.05 was considered statistically significant.

## Results

### Characterisation of hDPSCs

**Fig. 1:**
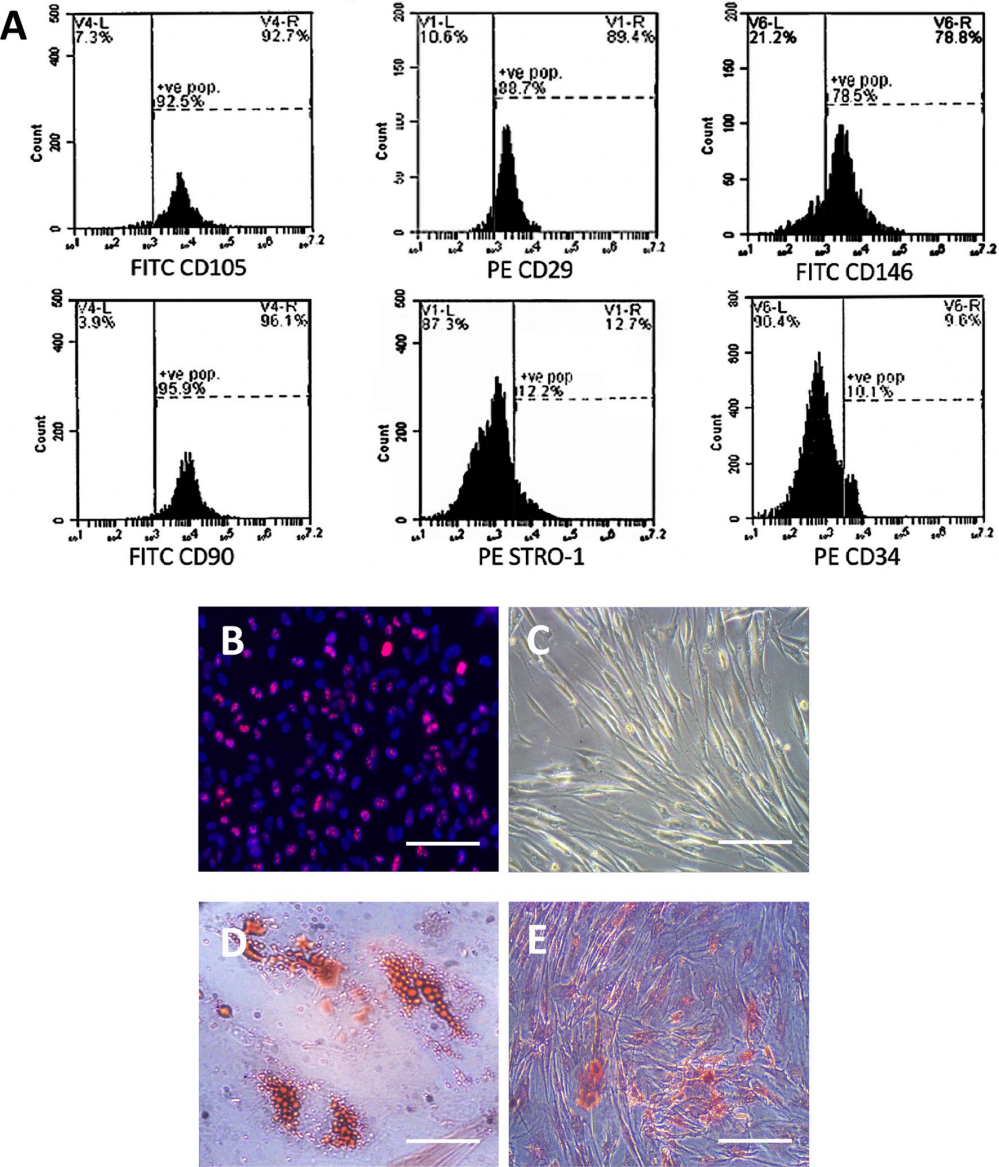
Characterisation of human dental pulp stem cells (hDPSCs). Cell surface expression of CD105, CD29, CD146, CD90, stromal precursor antigen (STRO-1), and CD34 was determined using flow cytometry (**A**); most of the cells express the KI67 marker (**B**); and exhibit a spindle-shaped morphology (**C**). In vitro multi-lineage differentiation potential of hDPSCs into adipocytes as identified by Oil-Red O staining (**D**) and osteocytes as identified by Alizarin Red S staining (**E**) is demonstrated after 2 weeks of culture in appropriate induction media. Scale bars = 100 μm

The hDPSCs from passage 4 were analysed for the expression of CD105, CD29, CD146, CD90, and STRO-1 (mesenchymal cells markers) and for the expression of CD34 (a haematopoietic lineage marker), using flow cytometry analysis. Of these cells, 92.5%, 88.7%, 78.5%, 95.9%, and 12.2% expressed CD105, CD29, CD146, CD90, and STRO-1, respectively, whereas 10.1% expressed CD34 (Fig. 1A). These results indicated that the isolated cells were mostly nonhaematopoietic MSCs. Most hDPSCs expressed KI67, indicating active proliferation (Fig. 1B), and exhibited fibroblast-like (spindle-shaped) morphology in culture (Fig. 1C). After 2 weeks of culture in appropriate induction media, hDPSCs demonstrated in vitro multi-lineage differentiation capacity into adipocytes, as identified with Oil-Red O staining (Fig. 1D), and into osteocytes as identified with Alizarin Red S staining (Fig. 1E).

### Transplanted hDPSCs attenuated hyperglycaemia in STZ-diabetic rats

**Fig. 2:**
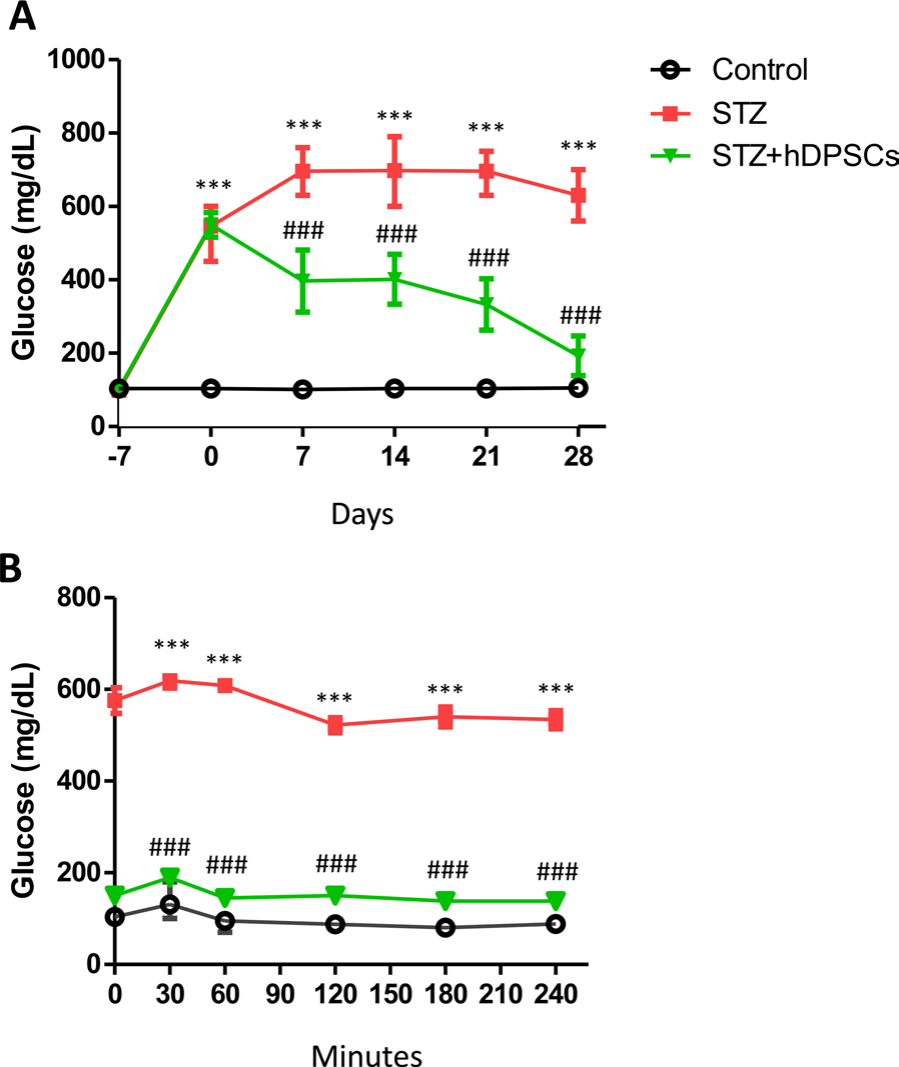
Blood glucose profiles of rats in the control, diabetic (streptozotocin [STZ]), and transplanted (STZ + human dental pulp stem cells [hDPSCs]) groups. **A** Blood glucose levels were monitored once weekly. **B** Glucose tolerance tests were performed 28 days post-transplantation; ***p < 0.001 compared with the control group; ^###^*p* < 0.001 compared with the STZ group. Data are expressed as the mean ± SEM. *n* = 10 per group

Prior to STZ injection, the blood glucose concentration of the rats was 103.8 ± 4 mg/dL. This increased to 546.0 ± 11 mg/dL 1 week after STZ treatment. To evaluate the effects of hDPSCs on blood glucose levels, cells were injected into the tail vein of STZ-treated rats. The blood glucose levels in the STZ + hDPSCs group dropped significantly within 7 days of transplantation (*p* < 0.001; Fig. 2A) and remained significantly lower than those of the diabetic group (STZ) at 21 days (*p* < 0.001; Fig. 2A) and 28 days (*p* < 0.001; Fig. 2A) after hDPSC transplantation.

To evaluate the effect of hDPSCs on glucose homeostasis, glucose tolerance tests were performed 28 days post-transplantation. After intraperitoneal glucose injections, the blood glucose levels of the STZ + hDPSCs group were significantly lower than those in the STZ group (*p* < 0.001; Fig. 2B). These results suggested that pancreatic function was restored and maintained 28 days after hDPSC transplantation in rat model of STZ-induced type I diabetes.

### Transplanted hDPSCs improved parotid gland atrophy and dysfunction

**Fig. 3:**
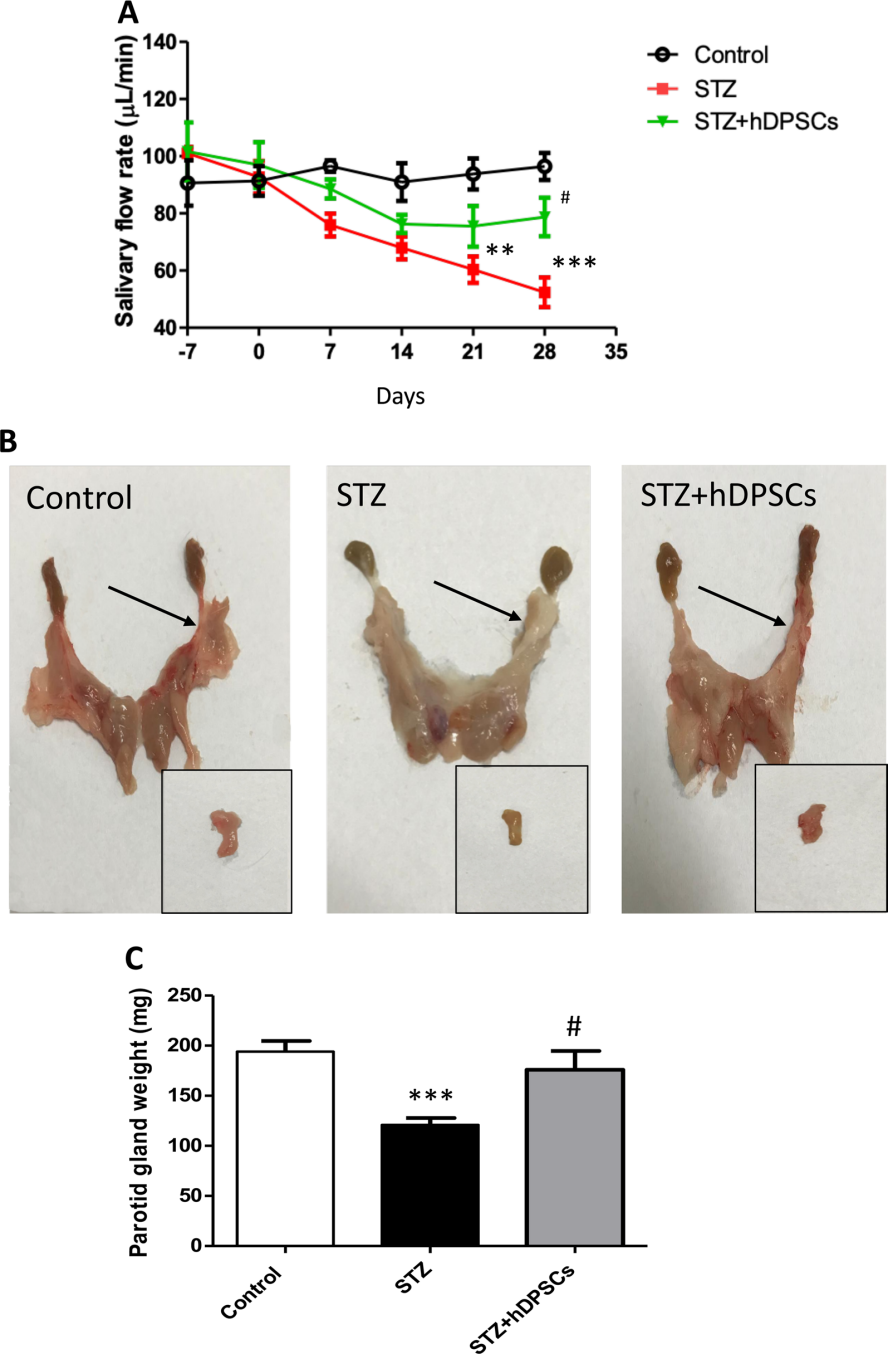
Salivary flow rate (**A**), macroscopic parotid tissue (arrows, **B**) shown after dissection (insets), and parotid gland weight (**C**), in the control, diabetic (streptozotocin [STZ]), and transplanted (STZ + human dental pulp stem cells [hDPSCs]) groups. ***p < 0.001, compared with the control group; and ^#^*p* < 0.05, compared with the STZ group. Data are expressed as the mean ± SEM. *n* = 10 for each group

The effects of STZ-induced diabetes on parotid gland atrophy and dysfunction in rats and the potential beneficial effects of hDPSC injection were investigated. A significant reduction in the total saliva output was detected in diabetic rats compared with that in the control group at 21 days (*p* < 0.01; Fig. 1A) and 28 days (*p* < 0.001; Fig. 1A) after STZ injection. However, saliva secretion significantly increased in rats in the STZ + hDPSCs group compared with that in the STZ group at 28 days (*p* < 0.05; Fig. 3A) after hDPSC transplantation. On day 28 post-transplantation, the parotid gland weight of STZ-treated rats was significantly lower as compared with that in the control rats (*p* < 0.001; Fig. 3B, C). Notably, the parotid glands of the STZ + hDPSCs group had a significantly higher weight compared to the diabetic group (*p* < 0.05; Fig. 3B, C). These results suggested that hDPSCs partially ameliorated diabetic-induced parotid gland atrophy and hyposalivation.

### Transplanted hDPSCs ameliorated the changes in antioxidant and oxidative stress indices in the STZ-treated parotid gland

**Fig. 4:**
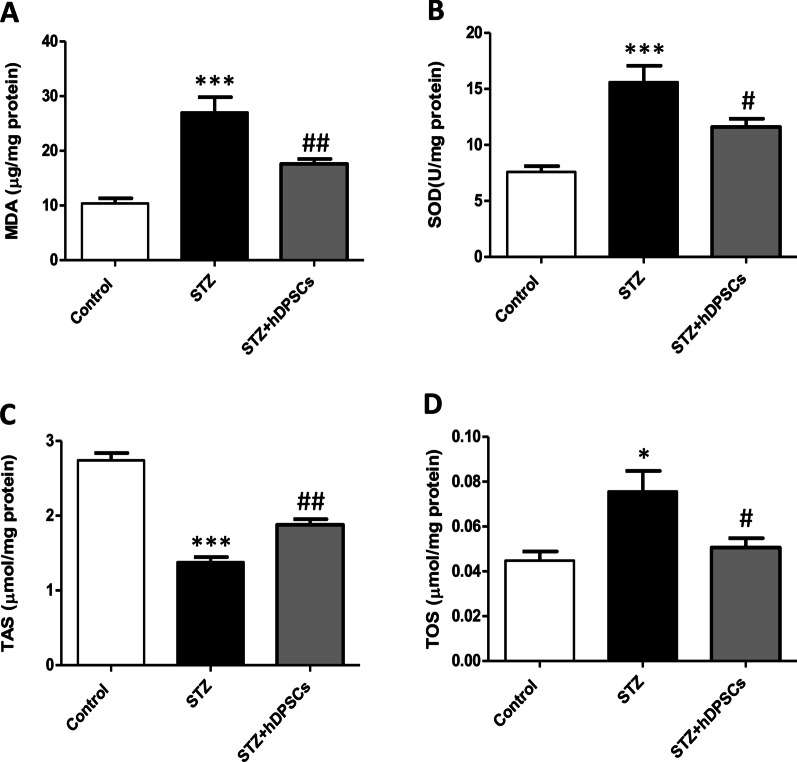
Levels of malondialdehyde (MDA, **A**), superoxide dismutase (SOD, **B**) enzymatic activity, total antioxidant capacity (TAS, **C**), and total oxidative status (TOS, **D**) in the control, diabetic (streptozotocin [STZ]), and transplanted (STZ + human dental pulp stem cells [hDPSCs]) groups. *p < 0.5 and ***p < 0.001, compared with the control group; ^#^*p* < 0.05 and ^##^*p* < 0.01, compared with the STZ group. Data are expressed as the mean ± SEM. *n* = 10 for each group

The parotid glands of STZ-treated rats had higher levels of MDA, an index of lipid peroxidation, compared to those of the control group (*p* < 0.001; Fig. 4A). In parallel, SOD was significantly increased in the parotid glands of STZ-treated rats compared with the levels of the control rats (*p* < 0.001; Fig. 4B). However, a significant reduction in the TAS level (*p* < 0.001; Fig. 4C) and a significant increase in the TOS level (*p* < 0.05; Fig. 4D) were observed in the parotid glands of diabetic rats compared with those in the parotid glands of the control group. In rats in the STZ + hDPSCs-treated group, significant reductions in MDA (*p* < 0.01; Fig. 4A) and SOD levels (*p* < 0.05; Fig. 4B) were observed compared with the levels in the STZ-treated rats. In addition, a significant increase in the TAS level (*p* < 0.01; Fig. 4C) and a significant decrease in the TOS level (*p* < 0.05; Fig. 4D) were observed in the parotid glands of rats in the STZ + hDPSCs-treated group compared with those in diabetic rats.

### Transplanted hDPSCs attenuated the histopathological changes in the STZ-treated parotid gland

**Fig. 5:**
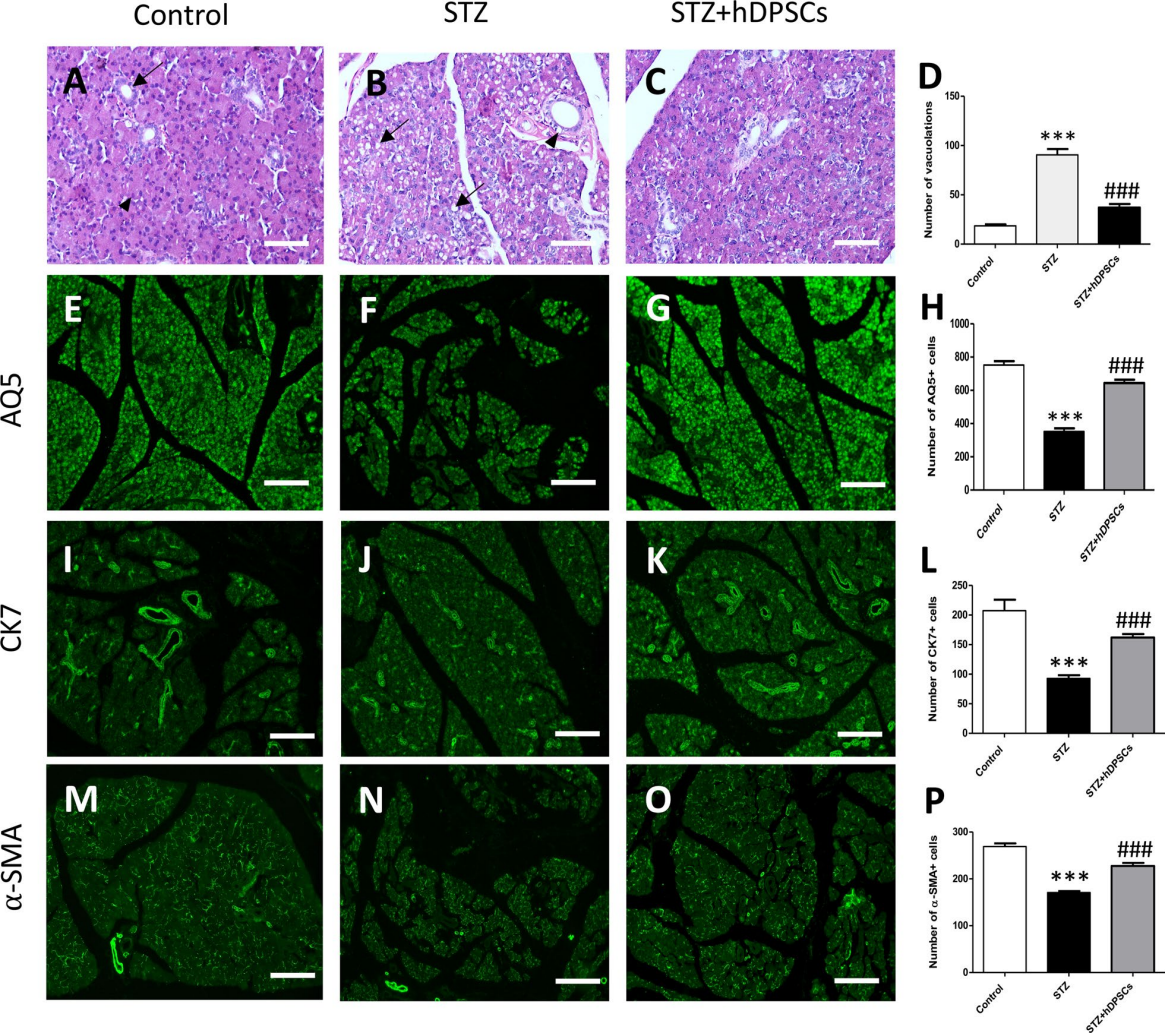
Effects of transplanted human dental pulp stem cells (hDPSCs) on different parotid cell subpopulations. Representative haematoxylin and eosin staining and number of vacuolations (**A**–**D**). Representative immunofluorescence staining for and number of aquaporin 5 (AQP5)- (**E**–**H**), cytokeratin 7 (CK7)- (**I**–**L**), and α-smooth muscle actin (α-SMA)- (**M**–**P**) positive cells in the control, diabetic (streptozotocin [STZ]), and transplanted (STZ + hDPSCs) groups. ***p < 0.001, compared with the control group; ^###^*p* < 0.001, compared with the STZ group. Data are expressed as means ± SEM. *n* = 10 for each group. Scale bars = 200 μm (**A**–**C**) and 500 μm (**E**–**G**, **I**–**K**, **M**–**O**)

To determine whether the preservation of salivary function in the transplanted group was related to an improvement in STZ-induced histopathological alterations in the parotid gland, we stained tissue sections with haematoxylin and eosin and observed that the parotid glands of the control group exhibited a normal glandular tissue architecture. The specimens displayed serous acini together with multiple striated ducts. The serous acini were lined with pyramidal cells with basal rounded nuclei (Arrow head, Fig. 5A), whereas the striated ducts were lined with columnar cells (Arrow, Fig. 5A). In the diabetic group, the parotid glands showed vacuolation of the acinar cells, indicating degenerative changes (*p* < 0.001; Arrows, Fig. 5B, D). In addition, the cells of striated ducts appeared atrophic with many vacuoles and pyknotic nuclei (Arrow head, Fig. 5B). In rats of the STZ + hDPSCs group, the number of the vacuoles in both the acinar and ductal cells were significantly reduced, and structural improvement were noted in the gland (*p* < 0.001; Fig. 5C, D). In addition, we used immunofluorescence to assess the expression levels of several proteins in parotid gland cell subpopulations. In STZ rats, a significantly lower number of cells expressed AQP5 (an acinar cell marker; *p* < 0.001; Fig. 5E–H), CK7 (a marker for ductal/progenitor cells; Fig. 5I–L), and α-SMA (a marker for myoepithelial cells; *p* < 0.001; Fig. 5M–P) compared with the control rats. Significantly higher expression of these markers was observed in rats in the STZ + hDPSCs group than in rats in the STZ group (*p* < 0.001; Fig. 5H, L, P).

**Fig. 6:**
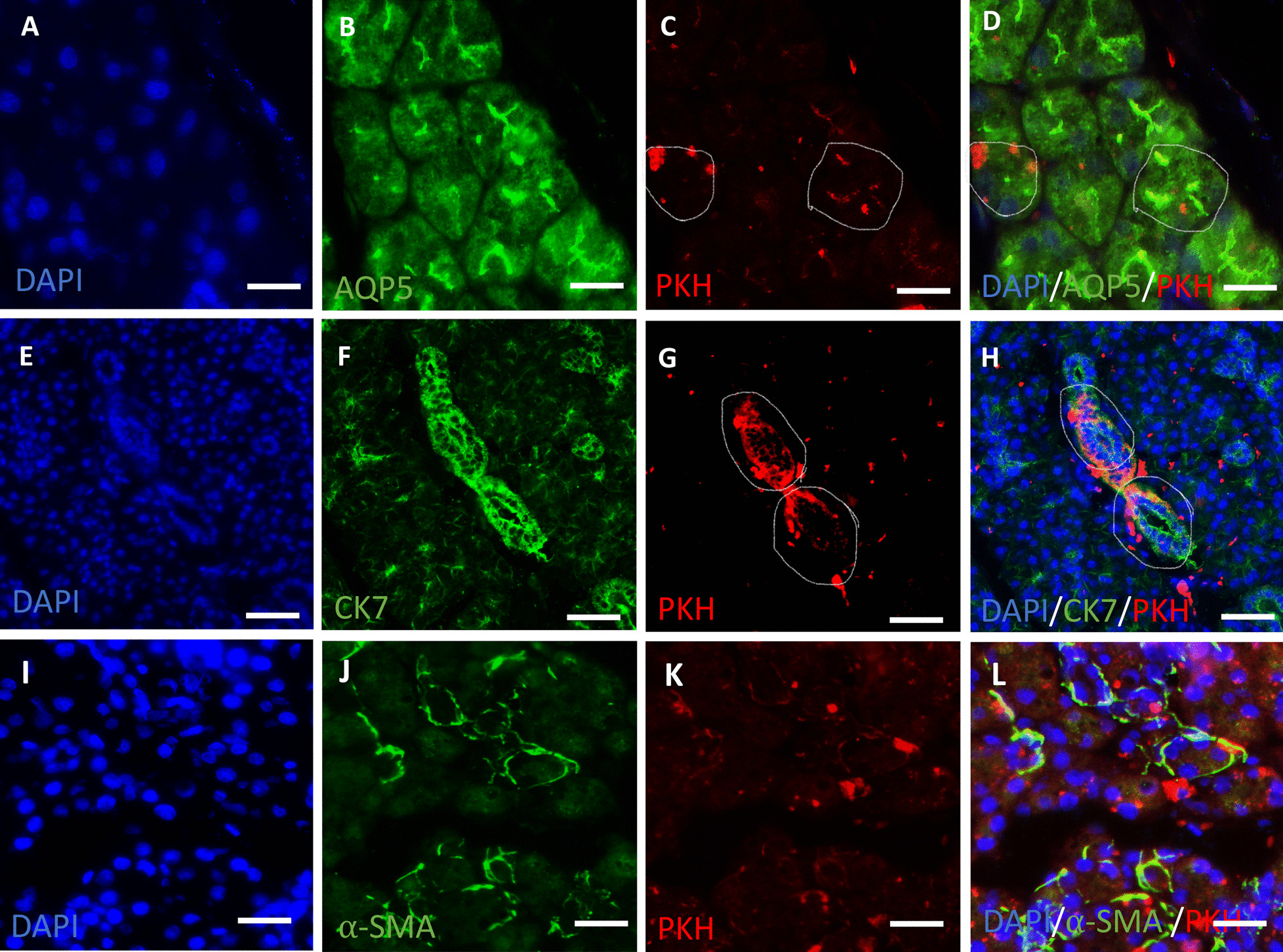
The survival and differentiation of transplanted human dental pulp stem cells (hDPSCs) into acinar, ductal, and myoepithelial cells in the transplanted (streptozotocin [STZ] + hDPSCs) group. Number of PKH26-labelled hDPSCs co-expressing aquaporin 5 (AQP5) (**A**–**D**), cytokeratin 7 (CK7) (**E**–**H**), and α-smooth muscle actin (α-SMA) (**I**–**L**). Some PKH26 + AQP5 and PKH26 + CK7 cells exhibited acinar and duct-like arrangements, denoted with a white line (**C**, **D**, **G**, **H**), suggesting cellular organisation into functional components. PKH26-labelled cells (red) **C**, **G**, **K**; AQP5-positive cells (green) **B**, CK7-positive cells (green) **F**, α-SMA-positive cells (green) **J**; DAPI-stained nuclei (blue) **A**, **E**, **I**. Merged images (**D**, **H**, **L**). Scale bar = 50 μm (**A**–**D** and **I**–**L**) and 75 μm (**E**–**H**)

To evaluate the engraftment of the transplanted hDPSCs into the parotid gland, we performed an immunofluorescence-based assessment of the parotid glands of the STZ + hDPSCs group 28 days post-transplantation. Transplanted hDPSCs were distinguished from recipient cells by their PKH26 label. Large numbers of PKH26-labelled cells were detected in the parotid glands of the STZ + hDPSCs group. Differentiation of the transplanted cells into glandular phenotypes in the recipient gland was indicated by the colocalisation of AQP5 (Fig. 6A–D), CK7 (Fig. 6E–H), and α-SMA (Fig. 6I–L) with PKH26-labelled cells. The percentages of PKH26-labelled cells that co-expressed AQP5, CK7, and α-SMA were 31.1% ± 2.7%, 15.3% ± 0.6%, and 7% ± 1.6%, respectively. Interestingly, some PKH26 + AQP5 and PKH26 + CK7 cells exhibited acinar and duct-like arrangements, suggesting cellular organisation into functional components (Fig. 6C, D, G, H).

### Transplanted hDPSCs promoted cell survival and angiogenesis, and protected the endogenous parotid cells in STZ‑diabetic rats

**Fig. 7:**
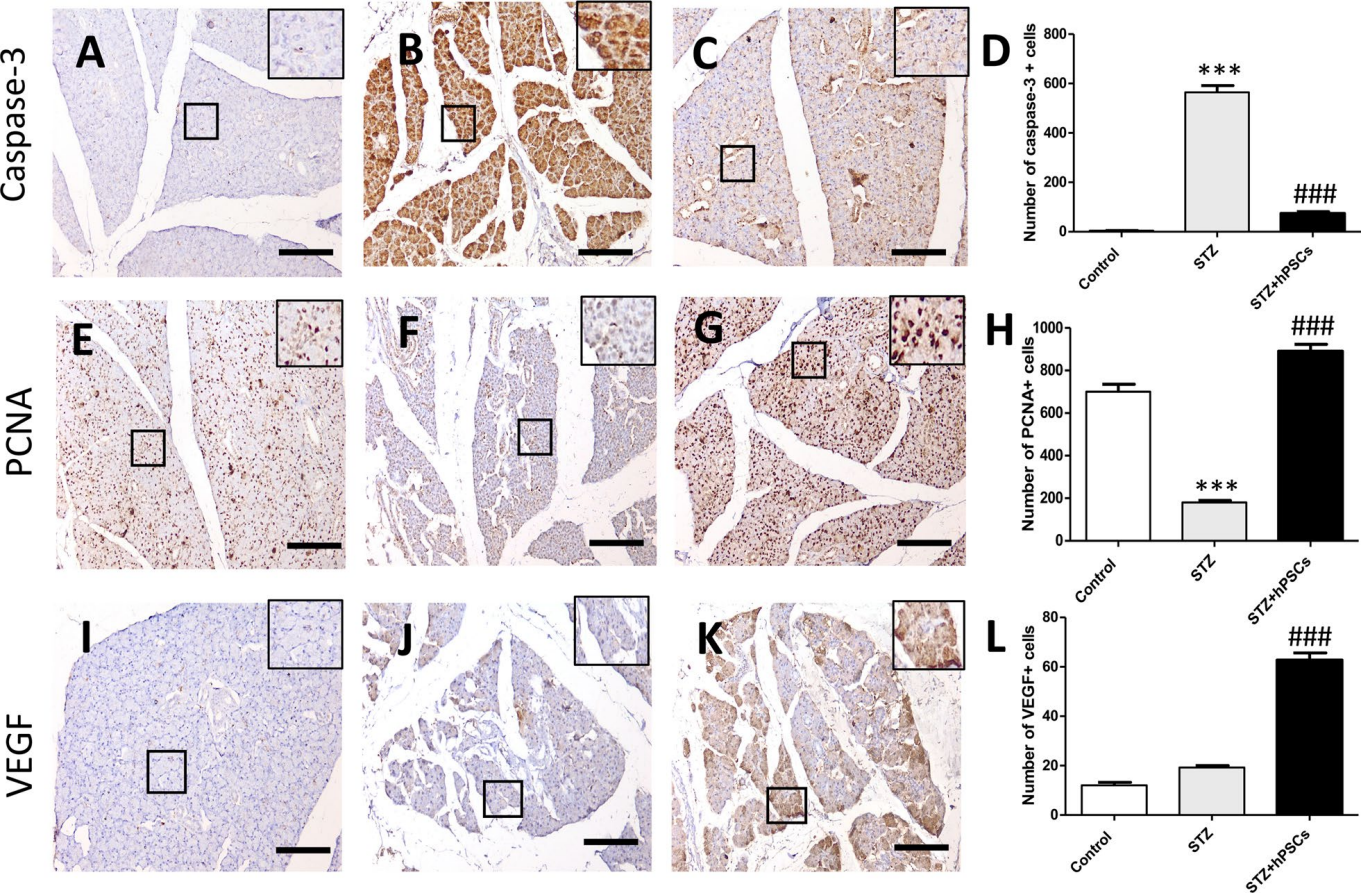
Effects of transplanted human dental pulp stem cells (hDPSCs) on the expression of caspase-3 (**A**–**D**), PCNA (**E**–**H**), vascular endothelial growth factor (VEGF) (**I**–**L**) in the control, diabetic (streptozotocin [STZ]), and transplanted (STZ + hDPSCs). The boxed areas appear at a higher magnification in the insets. ***p < 0.001 compared with the control group; ^###^*p* < 0.001 compared with the STZ group. Data are expressed as the mean ± SEM. n = 10 per group. Scale bars = 200 μm

To further explore the mechanisms through which the transplanted hDPSCs preserved the functional and morphological features of the parotid glands, we evaluated the potential anti-apoptotic, proliferative, and angiogenic effects of hDPSCs in the STZ + hDPSCs group. We observed that caspase-3 expression was upregulated, whereas the expression level of PCNA was significantly declined and that of VEGF was not significantly changed in the parotid gland of STZ diabetic rats compared with those in the control group (Caspase-3, *p* < 0.001, Fig. 7A, B; PCNA, *p* < 0.001, Fig. 7E, F). Interestingly, the parotid glands in the STZ + hDPSCs group had significantly reduced numbers of caspase-3-positive cells and increased numbers of PCNA- and VEGF-positive cells compared with those in diabetic rats (Caspase-3, *p* < 0.001, Fig. 7A–D; PCNA, *p* < 0.001, Fig. 7E–H; VEGF, *p* < 0.001, Fig. 7I–L), indicating that the transplanted cells likely exerted anti-apoptotic and angiogenic effects and protected the endogenous parotid cells.

### Transplanted hDPSCs upregulated neuronal NO synthase (nNOS), endothelial NO synthase (eNOS) gene expression and tetrahydrobiopterin (BH4) biosynthetic enzyme protein expression in the STZ-treated parotid gland

**Fig. 8:**
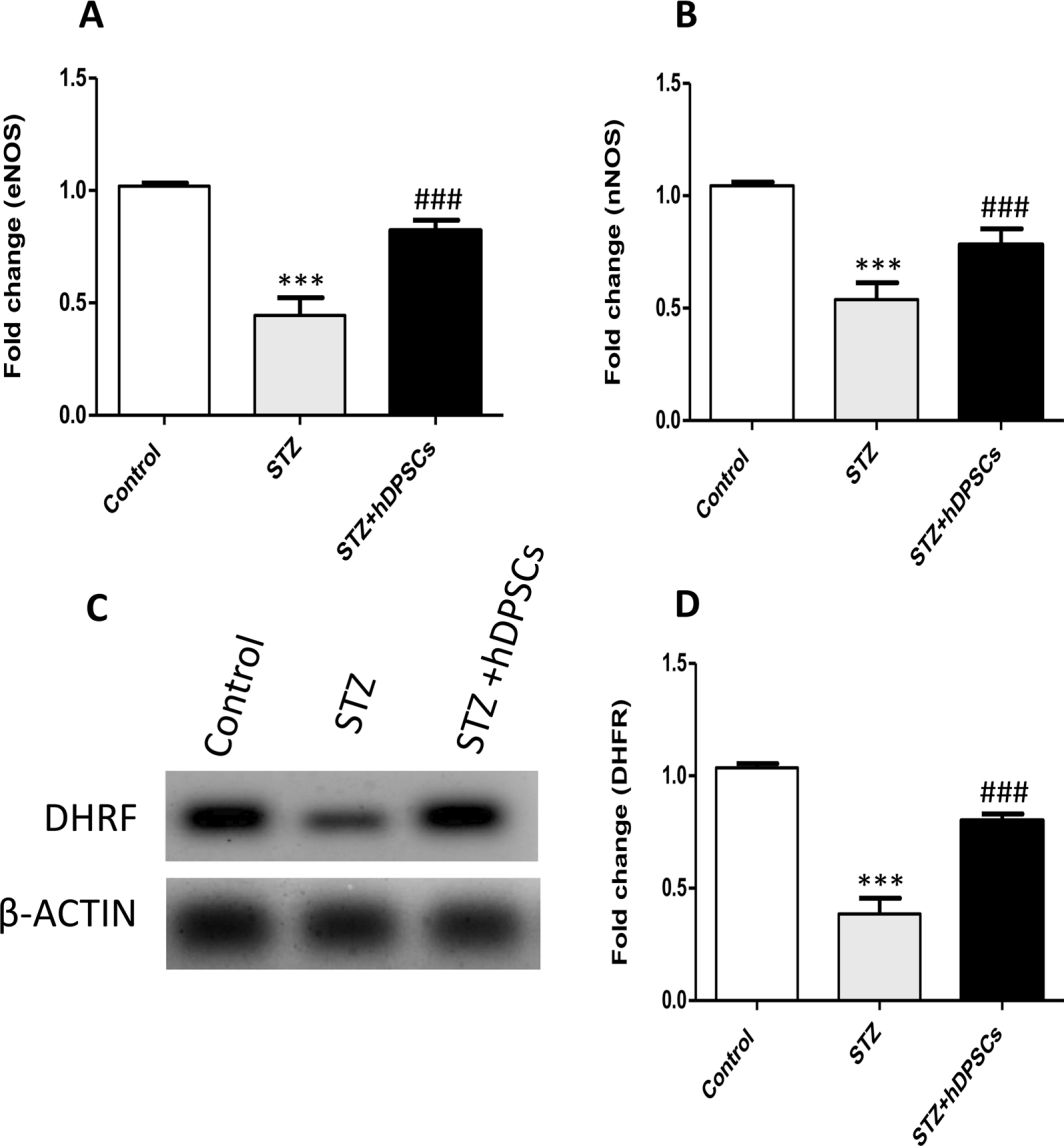
Effects of transplanted human dental pulp stem cells (hDPSCs) on the gene expression of neuronal nitric oxide synthase (nNOS) and endothelial neuronal nitric oxide synthase (eNOS) measured with RT-qPCR (**A** and **B**); and protein expression of DHFR (BH4 biosynthetic enzyme) measured with western blot (**C** and **D**) in the control, diabetic (streptozotocin [STZ]), and transplanted (STZ + hDPSCs) groups. ***p < 0.001 compared with the control group; ^###^*p* < 0.001 compared with the STZ group. Data are expressed as the mean ± SEM. *n* = 10 per group.

Gene expression of both *nNOS* and *eNOS* as well as protein expression of DHFR (BH4 biosynthetic enzyme) were significantly downregulated in the parotid glands of STZ diabetic animals compared with the parotid glands of control rats (*p* < 0.001; Fig. 8), while these expression levels were significantly increased in the parotid glands of the STZ + hDPSCs group compared with the parotid glands of diabetic rats (*p* < 0.001; Fig. 8).

## Discussion

Salivary hypofunction is a major concern for diabetic patients and has a major impact on patients’ quality of life [48]. SFR is one of the most important indices for the assessment of salivary gland function [4]. In our study, total salivary production was dramatically reduced in STZ rats compared with that in control rats. This was consistent with previous studies reporting STZ-induced hyposalivation [17, 49]. Furthermore, we detected a higher SFR in the hDPSC-injected group than in STZ rats. Consistent with this result, the parotid glands in the STZ + hDPSCs group had restored weight compared to those of the diabetic rats. Previous studies have reported that MSCs can structurally and functionally rescue salivary glands damaged by irradiation [24–26] and Sjögren’s syndrome [27–30]. However, to the best of our knowledge, this is the first study to show that hDPSC intravenous injection can partially rescue parotid gland atrophy and dysfunction in diabetic rats.

At 35 days post-STZ injection, a significant increase in MDA levels was detected, accompanied by a similar increase in SOD levels. Because the metabolism in the parotid gland is mainly aerobic, it is inherently prepared to counteract oxidative injury triggered by reactive oxygen species (ROS). Previous studies have reported that the parotid gland is mainly affected by hyperglycaemia during the initial stages of diabetes, showing a marked increase in lipid peroxidation and MDA levels. This is accompanied by an associated increase in SOD activity as an adaptive response to eradicate ROS [10, 19, 50]. Since the efficiency of the oxidative system cannot be evaluated by analysing only MDA and SOD levels, TOS and TAS levels were also assessed in the current study. In diabetic rats, a significant increase in TOS and a significant decrease in TAS were detected, indicating an enhancement of oxidative processes in the parotid gland and insufficient capacity of the antioxidant systems to eliminate excessive ROS. In contrast, in the parotid glands of the STZ + hDPSCs group, significant decreases in MDA and SOD levels, a significant increase in the TAS level, and a reduction in the TOS level were observed compared with those in the STZ-treated group, indicating the inhibitory effect of hDPSCs on oxidative stress. Our results are consistent with previous studies demonstrating the antioxidant abilities of DPSCs [51–53]. In addition, glucose is the main source of ROS production [54]. Our previous study demonstrated that hDPSCs can migrate to and survive within the STZ-injured pancreas and subsequently exert antidiabetic effects by differentiating into β-cells and inducing paracrine-mediated pancreatic regeneration [55]. Therefore, available scientific literature suggests that glycaemic control may play a role in hindering oxidative stress.

We showed that STZ injection promoted degenerative changes in the form of vacuolations in both acinar and ductal cells of parotid gland. These results were consistent with previous studies demonstrating that diabetes likely induces morphological alterations in salivary glands that are of a lipid nature as they are removed during the fixation and processing of the samples [10, 11, 13, 16–18]. In diabetes, vacuolations are more evident in the parotid gland than in the submandibular glands [10, 17]. The fatty degeneration is linked to diabetes-induced oxidative stress in the parotid glands and the associated apoptotic changes [16, 17, 20].

Our results revealed a decrease in different parotid gland cell populations, namely acinar, ductal, and myoepithelial cells in diabetic rats; these results were consistent with several earlier reports [29, 56, 57]. We detected a significant downregulation of AQP5, a key water channel protein located mainly on the apical membrane of salivary acinar cells, plays a major role in saliva secretion [13, 58, 59]. Many studies have demonstrated that downregulation and abnormal distribution of AQP5 in salivary glands account for disease-associated salivary hypofunction, as observed in Sjögren’s syndrome, following radiation exposure, and in non-obese patients with diabetes [60–63]. Our results showed that these histopathological changes were significantly attenuated by hDPSC injection. It is likely that the antioxidant and anti-apoptotic direct activities of hDPSCs protected and partially restored the normal architecture of the parotid gland.

To further elucidate the mechanisms underlying the structural and functional recovery observed in the parotid glands of the STZ + hDPSCs group, we investigated the differentiation potential of the transplanted hDPSCs. PKH26-labelled cells colocalised with AQ5, CK7, and α-SMA, indicating their differentiation into acinar, ductal, and myoepithelial cells, respectively. Previous studies have demonstrated that MSCs from different sources are capable of differentiating into acinar and ductal epithelial cells [24, 64]. Although the paracrine capability of hDPSCs has been demonstrated in different models of salivary hypofunction, the potential of these cells to differentiate into different types of salivary gland cells was not investigated in these studies [42–44]. To the best of our knowledge, this is the first study to demonstrate that DPSCs can differentiate into parotid acinar, ductal, and myoepithelial cells. Indeed, DPSCs, as the parotid glands, originate from the ectoderm, suggesting that these cells may have enhanced potential than other MSCs to differentiate into parotid cells.

In the current study, intravenously injected hDPSCs migrated to and survived in the parotid glands of diabetic rats and differentiated into parotid cells in the xenogeneic milieu. The survival of xenogeneic transplanted MSCs in immunocompetent adult recipients provides evidence of the immunoprivileged status of MSCs [65]. In addition, significant survival of the engrafted hDPSCs may be related to specific features of these cells. Previous studies have reported a superior immunosuppressive capacity of DPSCs compared with that of bone marrow MSCs. Huang et al. (2008) demonstrated that the implantation of DPSCs derived from rhesus monkeys into the hippocampi of mice did not induce immune rejection [66]. In another study, engraftment of hDPSCs into mouse livers led to the stimulation of human MHC-1 [67], suggesting strong immunosuppression [68].

The paracrine effects of DPSCs, via secretion of angiogenic and anti-apoptotic factors, and increasing expression of proliferation-promoting molecules, are thought to play key roles in tissue regeneration in many diseases, including those of the salivary gland [35, 69, 70]. Given the limited number of hDPSC derived-differentiated cells in our study and to further elucidate mechanisms through which the transplanted cells rescued the functional and structural changes in the diabetic parotid gland, we investigated the potential paracrine effects of the transplanted hDPSCs.

VEGF is a major growth factor and signalling molecule in angiogenesis and vasculogenesis. The hDPSCs possess high angiogenic capacity via their secretion of several angiogenic factors, including VEGF [71, 72]. Our results showed enhanced VEGF expression in the parotid glands of the hDPSC-treated rats compared with the untreated rats, suggesting that hDPSCs promote parotid gland regeneration via angiogenesis and neovascularisation, and hence, alleviate the microvascular alterations associated with diabetes. Our results were consistent with those of previous studies demonstrating the ability of hDPSCs to induce angiogenesis and increase acinar cell numbers to rescue submandibular gland defects in diabetic Wistar rats [42, 43]. The hDPSCs have been shown to contribute to the formation of salisphere-like structures when co-cultured with HSG cells in either Matrigel or hyaluronic acid hydrogel scaffolds. This was achieved through their angiogenetic effect, as indicated by the higher expression of murine endothelial and angiogenic markers such as Vegfr-3 and Vegf-C, in co-cultures of DPSCs + HSG as compared with that in HSG cultures alone [41].

Substantial evidence has demonstrated that adult salivary glands harbour a variety of epithelial and mesenchymal stem/progenitor populations. Under stress and injury conditions, salivary gland damage can be repaired by these cell populations, as they have proliferation potential and are capable of differentiating into both acinar and duct cells. However, in chronic illness conditions such as diabetes mellitus, the proliferation of these cells is largely affected, restricting their ability to compensate for the damage, resulting in the deterioration of the salivary function [73]. Our results showed that there was a significant decline in the numbers of PCNA-positive cells in the parotid glands of diabetic rats, suggesting an exhaustion of the proliferative capacity of the endogenous stem/progenitor cells, whereas the parotid glands of the transplanted rats had significantly increased numbers of these cells compared to the diabetic rats. Our results were consistent with several studies demonstrating the ability of different MSC sources or their extracts to promote cell proliferation in salivary glands after injury mediated by radiation [74–76] and Sjögren’s syndrome-like disease [29]. The high levels of proliferation detected in the parotid glands of the transplanted rats might indicate ongoing endogenous repair by the stem/progenitor populations, which are protected and enhanced by the local paracrine-mediated actions of the transplanted hDPSCs.

In addition, a balance between cell proliferation and apoptosis is required for salivary gland homeostasis [77–79]. In the diabetic rats, the parotid glands exhibited increased apoptosis, while this increase was suppressed in the parotid gland of the transplanted rats, as indicated by caspase-3 immunostaining. The results of the present study demonstrated the role of MSCs in inducing antiapoptotic effects in salivary gland injury, and are in accordance with previous reports [24, 25, 80, 81]. Our results suggest that hDPSCs likely mediate further parotid repair and regeneration by secreting anti-apoptotic factors, including VEGF [82].

Stewart et al. [83] reported that a decline in submandibular NO-BH_4_ protein expression may have a role in the development of hyposalivation in diabetes-induced xerostomia. Nitric oxide (NO), synthesised by a family of enzymes known as NO synthases, such as nNOS and eNOS, has been reported to be an essential signalling molecule in normal salivary gland function. NO is a potent vasodilator, has a major impact on angiogenesis, and also acts as a neurotransmitter [84]. Enzymatic uncoupling of NOS due to lack of BH_4_ may lead to a decrease in NO production and stimulate oxidative stress [85]. Our results demonstrated that transplanted hDPSCs upregulated the declined gene expression of *nNOS* and *eNOS* and protein expression of DHFR (BH_4_ biosynthetic enzyme) in the parotid glands of diabetic rats, suggesting that hDPSCs likely promoted angiogenesis and reduced oxidative stress via enhancing the NO-BH_4_ pathway.

Our study had some limitations. Since the hypo-immunogenicity of MSCs is well established due to their lack of MHC II expression and low levels of MHC I expression [32], no immunosuppressive agent was administered to xenografted rats in this study. Our results showed that the transplanted hDPSCs exhibited high survival rates at 28 days post-transplantation. However, as the results of several studies have suggested, an immune response at later time points could eventually lead to immunorejection [66, 86]. It is plausible that the administration of immunosuppressants and anti-inflammatory agents may further improve graft survival and enhance the differentiation potential of these cells in vivo, supporting their use in clinical settings in the future. Another limitation is that we only investigated the therapeutic efficacy of undifferentiated hDPSCs. Indeed, assessing the therapeutic outcome of undifferentiated and hDPSC-derived parotid subpopulations would help us gain a deeper understanding of the mechanisms underlying the action of these cells.

## Conclusions

Our results show that intravenous transplantation of hDPSCs rescued parotid gland injury in a rat model of STZ-induced type 1 diabetes. Our results provide evidence for the use of systemically transplanted hDPSCs as a potential therapeutic approach for the treatment of patients with diabetes-induced parotid injury. However, prior to commencing clinical trials, further studies are needed to optimise the differentiation of these cells into parotid subpopulations in vivo and to further understand the molecular mechanisms underlying the beneficial effects of hDPSC-secreted factors.

## Supplementary Information

**Additional file 1: Figure S1**. Reliability of PKH26 labelling. Most of PKH labelled cells co-express human nuclei antibody (HNA, arrows in B). PKH labelling is localised in the cytoplasm (red), whereas HNA staining is localised in the nucleus (green). DAPI-stained nuclei (blue, A); PKH26-labelled cells (red, B); HNA-stained nuclei (green, B). Merged image (C). The boxed areas appear at a higher magnification in the insets. Scale bar = 500 μm. **Figure S2.** Specificity of the antibody against aquaporin 5 (AQ5) used in the present study. A) Negative control (the primary antibody omitted) demonstrating no expression. B) Representative immunostaining for AQ5 in rat salivary tissue sample from the control group showing the characteristic localisation of AQ5 protein at the apical membrane and the basolateral membrane of the acinar cells (arrow heads), while no immunostaining can be seen in the ducts (arrow). C) Rat brain tissue was used as a negative tissue control, showing no expression. Scale bar = 100 μm (A and B) and 500 μm (C). **Figure S3.** Specificity of the antibody against cytokeratin 7 (CK7) used in the present study. A) Negative control (the primary antibody omitted) demonstrating no expression. B) Representative immunostaining for CK7 in rat salivary tissue sample from the control group showing the ductal cells exhibiting strong CK7 immunostaining (arrow), while weak expression can be detected in the acinar cells (arrow head). C) Rat brain tissue was used as a negative tissue control, showing no expression. Scale bar = 100 μm (A and B) and 500 μm (C). **Figure S4.** Specificity of the antibody against α smooth muscle actin (α-SMA) used in the present study. A) Negative control (the primary antibody omitted) demonstrating no expression. B) Representative immunostaining for α-SMA in rat salivary tissue sample from the control group showing α-SMA-positive myoepithelial cells with thin, branching processes are seen wrapping the acini and intercalated ducts (arrow heads), while no immunostaining can be seen in the ducts (arrow). C) α-SMA immunostaining in blood vessel walls served as an internal positive control for the specificity of the antibody (arrows). Scale bar = 100 μm. **Figure S5.** Specificity of the antibody against vascular endothelial growth factor (VEGF) used in the present study. A) Negative control (the primary antibody omitted) demonstrating no expression. B) Representative immunostaining for VEGF in rat salivary tissue sample from the STZ + hDPSCS group. C) Rat liver tissue from a hepatocellular carcinoma model was used as a positive tissue control. Scale bar = 100 μm (A and B) and 50 μm (C). **Figure S6.** Specificity of the antibody against PCNA used in the present study. A) Negative control (the primary antibody omitted) demonstrating no expression. B) Representative immunostaining for PCNA in rat salivary tissue sample from the STZ + hDPSCS group. C) Rat liver tissue from a hepatocellular carcinoma model was used as a positive tissue control. Scale bar = 100 μm (A and B) and 50 μm (C). **Figure S7.** Specificity of the antibody against caspase-3 used in the present study. A) Negative control (the primary antibody omitted) demonstrating no expression. B) Representative immunostaining for caspase-3 in rat salivary tissue sample from the STZ + hDPSCS group. C) Rat colon tissue sample from an acetic acid-induced ulcerative colitis model was used as a positive tissue control. Scale bar = 100 μm (A and B) and 200 μm (C)

## Data Availability

The datasets generated and/or analysed during the current study are available from the corresponding author on reasonable request.

## Abbreviations

ABTS: 2,2′-Binamine-di-3-ethylbenzothiazolin-6-sulphonic acid
α-SMA: Alpha (α)-smooth muscle actin
AQP5: Aquaporin 5
BH4: Tetrahydrobiopterin
CK7: Cytokeratin 7
DAPI: 4′,6-Diamidino-2-phenylindole
DHFR: Dihydrofolate reductase
DMEM: Dulbecco’s modified Eagle medium
DPSCs: Dental pulp stem cells
eNOS: Endothelial nitric oxide synthase
FBS: Foetal bovine serum
hDPSCs: Human dental pulp stem cells
HSG: Human salivary gland
MDA: Malondialdehyde
MHC: Major histocompatibility complex
MSCs: Mesenchymal stem cells
nNOS: Neuronal nitric oxide synthase
NO: Nitric oxide
PBS: Phosphate-buffered saline
PCNA: Proliferating cell nuclear antigen
ROS: Reactive oxygen species
RT: Room temperature
SFR: Salivary flow rate
SOD: Superoxide dismutase
STRO-1: Stromal precursor antigen-1
STZ: Streptozotocin
TAS: Total antioxidant capacity
TBS: Tris-buffered solution
TOS: Total oxidative status
VEGF: Vascular endothelial growth factor
