# Supplementary material for: Human dental pulp stem cells attenuate streptozotocin-induced parotid gland injury in rats
Source: Stem Cell Res Ther. 2021 Nov 14;12:577. doi: 10.1186/s13287-021-02646-6 (PMC8591949; doi:10.1186/s13287-021-02646-6)
Supplement: Supplementary file 1 — Additional file 1: Figure S1. Reliability of PKH26 labelling. Most of PKH labelled cells co-express human nuclei antibody (HNA, arrows in B). PKH labelling is localised in the cytoplasm (red), whereas HNA staining is localised in the nucleus (green). DAPI-stained nuclei (blue, A); PKH26-labelled cells (red, B); HNA-stained nuclei (green, B). Merged image (C). The boxed areas appear at a higher magnification in the insets. Scale bar = 500 μm. Figure S2. Specificity of the antibody against aquaporin 5 (AQ5) used in the present study. A) Negative control (the primary antibody omitted) demonstrating no expression. B) Representative immunostaining for AQ5 in rat salivary tissue sample from the control group showing the characteristic localisation of AQ5 protein at the apical membrane and the basolateral membrane of the acinar cells (arrow heads), while no immunostaining can be seen in the ducts (arrow). C) Rat brain tissue was used as a negative tissue control, showing no expression. Scale bar = 100 μm (A and B) and 500 μm (C). Figure S3. Specificity of the antibody against cytokeratin 7 (CK7) used in the present study. A) Negative control (the primary antibody omitted) demonstrating no expression. B) Representative immunostaining for CK7 in rat salivary tissue sample from the control group showing the ductal cells exhibiting strong CK7 immunostaining (arrow), while weak expression can be detected in the acinar cells (arrow head). C) Rat brain tissue was used as a negative tissue control, showing no expression. Scale bar = 100 μm (A and B) and 500 μm (C). Figure S4. Specificity of the antibody against α smooth muscle actin (α-SMA) used in the present study. A) Negative control (the primary antibody omitted) demonstrating no expression. B) Representative immunostaining for α-SMA in rat salivary tissue sample from the control group showing α-SMA-positive myoepithelial cells with thin, branching processes are seen wrapping the acini and intercalated ducts (arrow heads), whi [file 13287_2021_2646_MOESM1_ESM.pdf]

Additional File 1

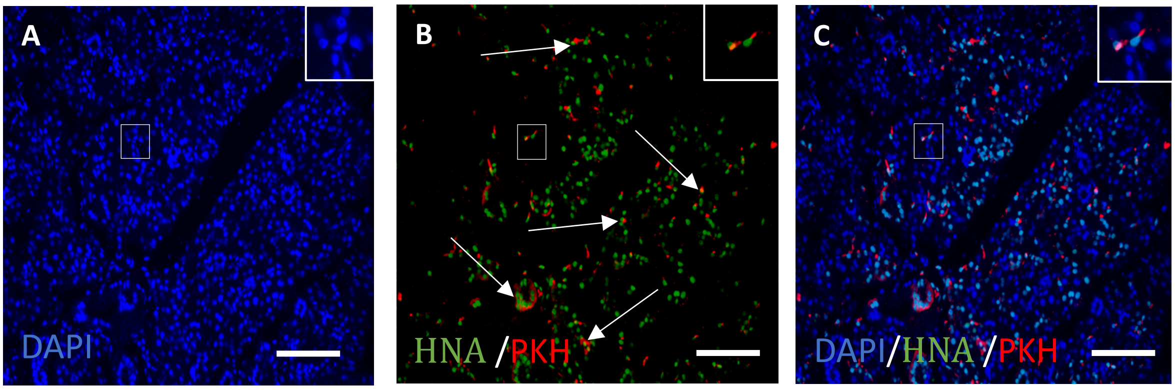

Figure S1. Reliability of PKH26 labelling. Most of PKH labelled cells co-express human nuclei antibody (HNA, arrows in B). PKH labelling is localized in the cytoplasm (red), whereas HNA staining is localized in the nucleus (green). DAPI-stained nuclei (blue, A); PKH26-labelled cells (red, B); HNA-stained nuclei (green, B). Merged image (C). The boxed areas appear at a higher magnification in the insets. Scale bar = 500 µm.

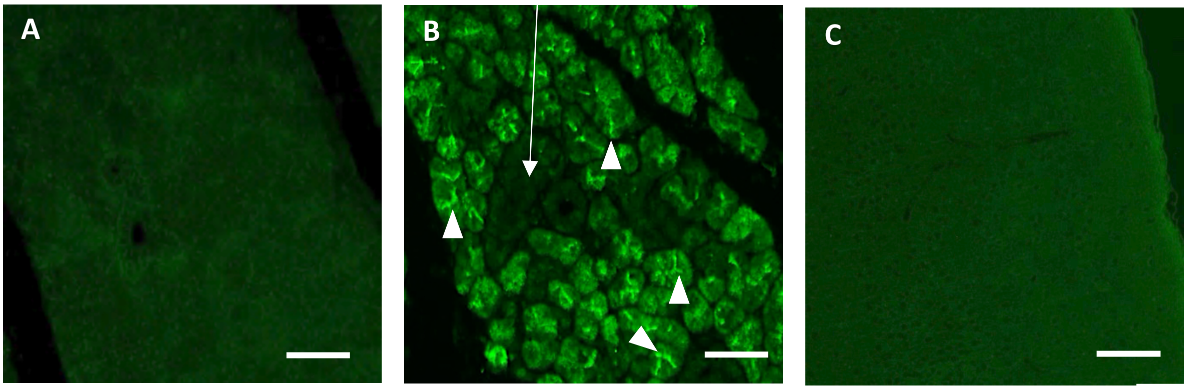

Figure S2. Specificity of the antibody against aquaporin 5 (AQ5) used in the present study. A) Negative control (the primary antibody omitted) demonstrating no expression. B) Representative immunostaining for AQ5 in rat salivary tissue sample from the control group showing the characteristic localization of AQ5 protein at the apical membrane and the basolateral membrane of the acinar cells (arrow heads), while no immunostaining can be seen in the ducts (arrow). C) Rat brain tissue was used as a negative tissue control, showing no expression. Scale bar = 100 µm (A and B) and 500 µm (C).

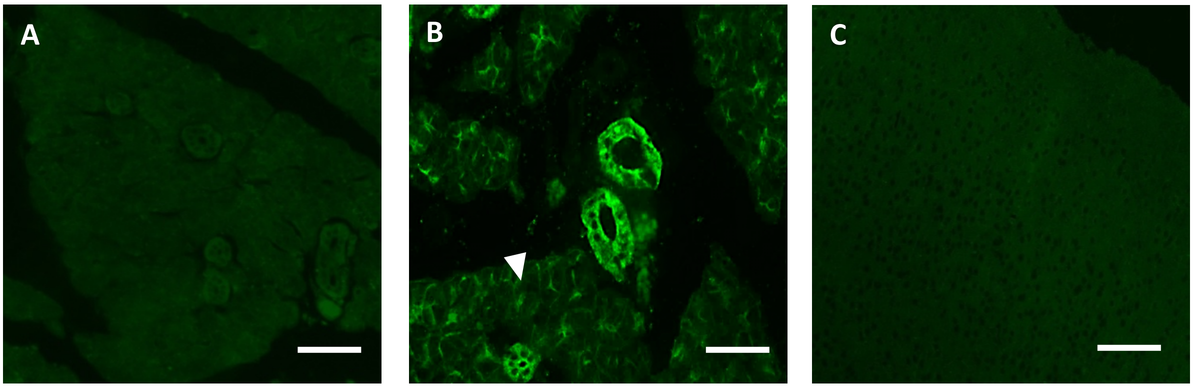

Figure S3. Specificity of the antibody against cytokeratin 7 (CK7) used in the present study. A) Negative control (the primary antibody omitted) demonstrating no expression. B) Representative immunostaining for CK7 in rat salivary tissue sample from the control group showing the ductal cells exhibiting strong CK7 immunostaining (arrow), while weak expression can be detected in the acinar cells (arrow head). C) Rat brain tissue was used as a negative tissue control, showing no expression. Scale bar = 100 µm (A and B) and 500 µm (C).

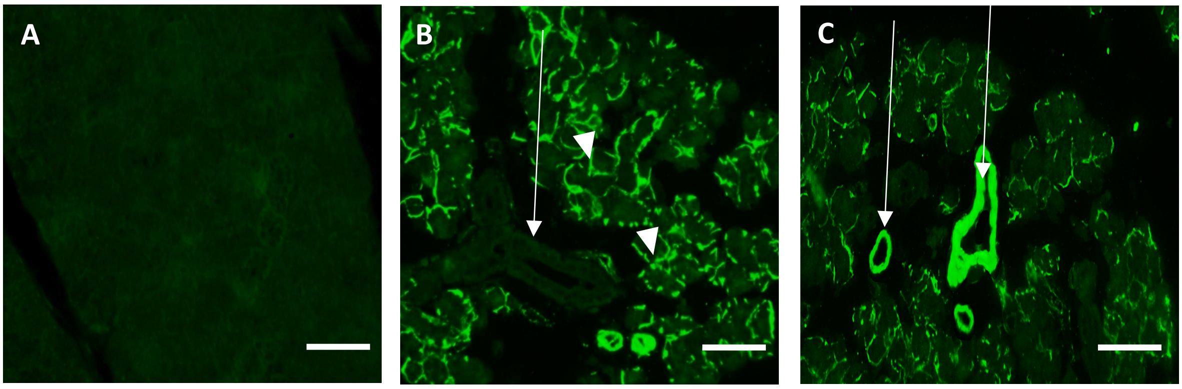

Figure S4. Specificity of the antibody against α smooth muscle actin (α-SMA) used in the present study. A) Negative control (the primary antibody omitted) demonstrating no expression. B) Representative immunostaining for α-SMA in rat salivary tissue sample from the control group showing α-SMA-positive myoepithelial cells with thin, branching processes are seen wrapping the acini and intercalated ducts (arrow heads), while no immunostaining can be seen in the ducts (arrow). C) α-SMA immunostaining in blood vessel walls served as an internal positive control for the specificity of the antibody (arrows). Scale bar = 100 μm.

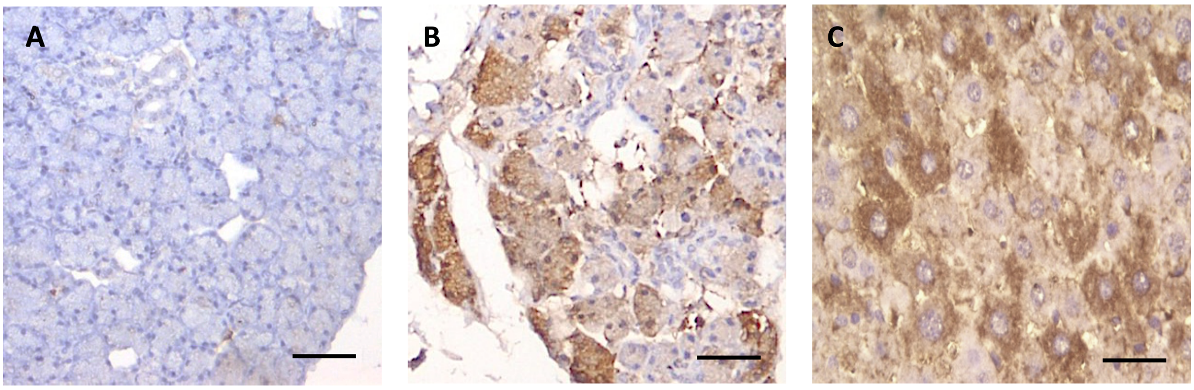

Figure S5. Specificity of the antibody against vascular endothelial growth factor (VEGF) used in the present study. A) Negative control (the primary antibody omitted) demonstrating no expression. B) Representative immunostaining for VEGF in rat salivary tissue sample from the STZ+hDPSCS group. C) Rat liver tissue from a hepatocellular carcinoma model was used as a positive tissue control. Scale bar = 100 μm (A and B) and 50 μm (C).

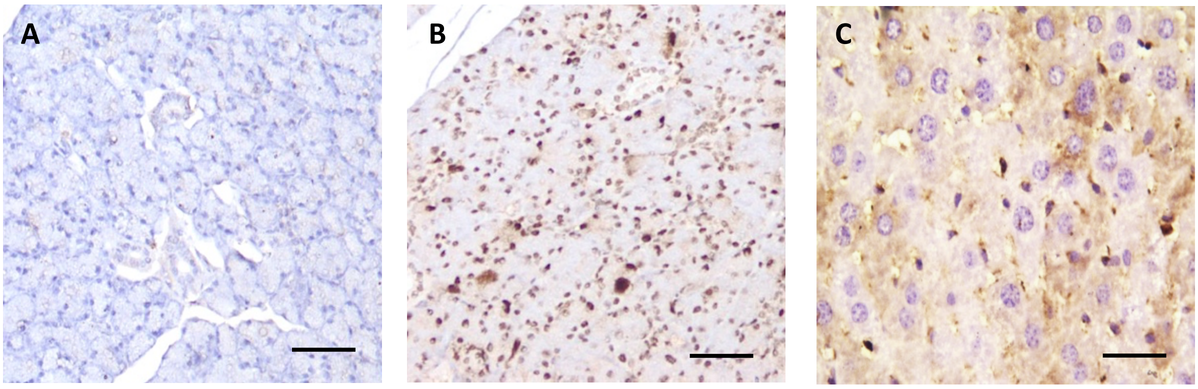

Figure S6. Specificity of the antibody against PCNA used in the present study. A) Negative control (the primary antibody omitted) demonstrating no expression. B) Representative immunostaining for PCNA in rat salivary tissue sample from the STZ+hDPSCS group. C) Rat liver tissue from a hepatocellular carcinoma model was used as a positive tissue control. Scale bar = 100 μm (A and B) and 50 μm (C).

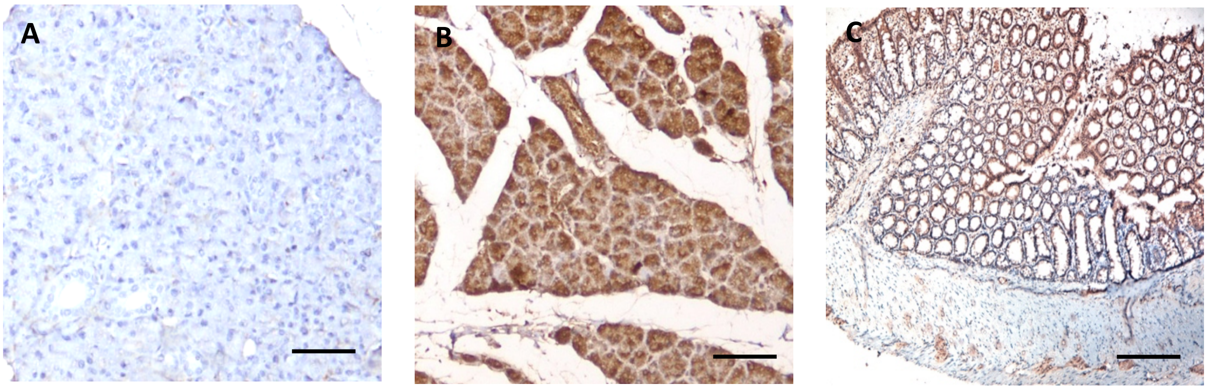

Figure S7. Specificity of the antibody against caspase-3 used in the present study. A) Negative control (the primary antibody omitted) demonstrating no expression. B) Representative immunostaining for caspase-3 in rat salivary tissue sample from the STZ+hDPSCS group. C) Rat colon tissue sample from an acetic acid-induced ulcerative colitis model was used as a positive tissue control. Scale bar = 100 μm (A and B) and 200 μm (C).
